# Bladder Leiomyoma in a 15-Year-Old Female Patient: A Case Report

**DOI:** 10.7759/cureus.61310

**Published:** 2024-05-29

**Authors:** Yunus Emre Genc, Naif Dinc Ulker, Muhammed Hasan Toper, Selcuk Yucel, Tarik Emre Sener

**Affiliations:** 1 Department of Urology, Marmara University School of Medicine, Istanbul, TUR; 2 Department of Pathology, Marmara University School of Medicine, Istanbul, TUR; 3 Department of Urology, Pediatric Urology Division, Marmara University School of Medicine, Istanbul, TUR

**Keywords:** treatment, transurethral resection of bladder, pediatric, leiomyoma, urinary bladder neoplasms

## Abstract

Bladder leiomyoma is a rare condition in pediatric and adolescent age groups, accounting for less than 1% of all bladder tumors, presenting a diverse array of histologic types and prevalence. Furthermore, bladder leiomyoma's prevalence is even more seldom with only five reported cases till the present day. Common presentation depends on the localisation and the affected layer in the bladder, urinary outlet or ureteral obstruction, irritative voiding symptoms, pelvic pain, and hematuria are the most common presentations of this condition. Diagnostic, treatment, and follow-up protocols in this entity are not well-established due to their rare occurrence in this age group. After complete surgical excision, the prognosis is excellent and the risk of recurrence is reported to be very low. Up to the present day, no instances of malignant transformation or metastasis have been documented in the literature.

This case report aims to enhance current knowledge of the radiological, pathological, and clinical features of bladder leiomyoma in a 15-year-old female patient. The main complaint was lower urinary tract symptoms. An incidental solid bladder mass was discovered during the evaluation with ultrasound and magnetic resonance imaging (MRI). Afterwards, cystoscopy confirmed a 5-centimeter solid mass at the right wall of the bladder, and transurethral piecemeal resection was performed. The bladder mass was found to be intramural, and complete endoscopic resection was considered safe and efficient during the surgery. No complications or recurrence occurred in the postoperative setting.

## Introduction

The prevalence and histologic types of bladder tumors may vary among paediatric, adolescent and adult age groups. Approximately 95% of primary bladder tumors arise from the urothelium whereas mesodermal tumors are previously reported for 5% of all neoplasms of the bladder in adults. Contradictory to adults, in children, urothelial neoplasms are rare and the most prominent tumors are papillary urothelial neoplasm of low malignant potential and rhabdomyosarcoma with a male predominance with a ratio of 2:1 [1].

The most common benign mesodermal bladder tumor is leiomyoma, which accounts for one-third of this type of tumor and accounts for less than 1% of all bladder tumor subtypes with a female predominancy [2], and additionally, its prevalence is extremely rare in children or adolescents with five reported cases to date [3-6].

The aetiology is poorly understood given their rarity, as these tumors can grow during pregnancy and regress postpartum, suggesting they are hormonally sensitive [5].

Common presentations of bladder leiomyomas include urinary outlet or ureteral obstruction, lower urinary tract symptoms (LUTS), pelvic pain, and hematuria. Furthermore, some of these cases have been diagnosed incidentally [7].

For initial assessment, ultrasonography and less often magnetic resonance imaging (MRI) can be used to better characterize lesions. On MRI, they are well-circumscribed and usually enhance after contrast administration. They are usually isointense to muscle on T1 and hypointense on T2 weighted images [8].

Discrimination among them is only possible by cystoscopy and histopathological examination due to a lack of their unique features in imaging modalities [8,9].

Cystoscopically they appear as bulging submucosal large-based lesions without ulceration and are usually covered by normal-looking mucosa on the surface, depending on the origin and localisation of the tumor [10].

While they might be located in several locations such as extravesical, intramural, or submucosal, the majority of cases, ranging from 63% to 86%, mostly occur in the submucosal layer, while extravesical and intramural leiomyomas account for 11% to 30% and 3% to 7% of cases, respectively [11].

The diagnostic, treatment and follow-up protocols in pediatric patients are not well-established due to their rare occurrence in this age group. As a result, there is a lack of evidence in the literature that provides clear recommendations for the management of this condition.

Some studies stated that after endoscopic, open or minimally invasive surgical resection, the prognosis is excellent and the risk of recurrence is very low. Therefore a routine follow-up is not recommended until lower urinary tract symptoms occur [11,12].

This case report seeks to enhance the current knowledge with radiological, pathological and clinical features of bladder leiomyoma in a 15-year-old female patient by following CARE (CAse REports) guidelines [13]. Written informed consent was obtained from the patient for publication.

## Case presentation

A 15-year-old female patient was admitted to our pediatric urology outpatient clinic with a weak urine stream, incomplete emptying, urgency and frequency for the last month.

Her past medical history was non-remarkable and physical examination revealed a normal urethra and external genitalia. Pelvic, abdominal and neurological examinations were also normal.

Ultrasound for evaluation of LUTS showed a heterogenous mildly lobulated iso-hypoechoic 40x46 mm solid mass at the right anterolateral wall of the urinary bladder. The serum creatinine level was 0.84 mg/dL, urine analysis was negative for haematuria or pyuria and the urine culture was sterile.

**Figure 1:**
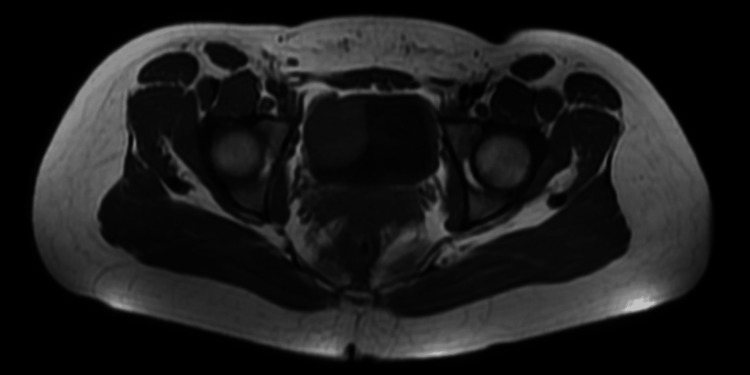
Pre-operative contrast-enhanced pelvic MRI T1 axial

**Figure 2:**
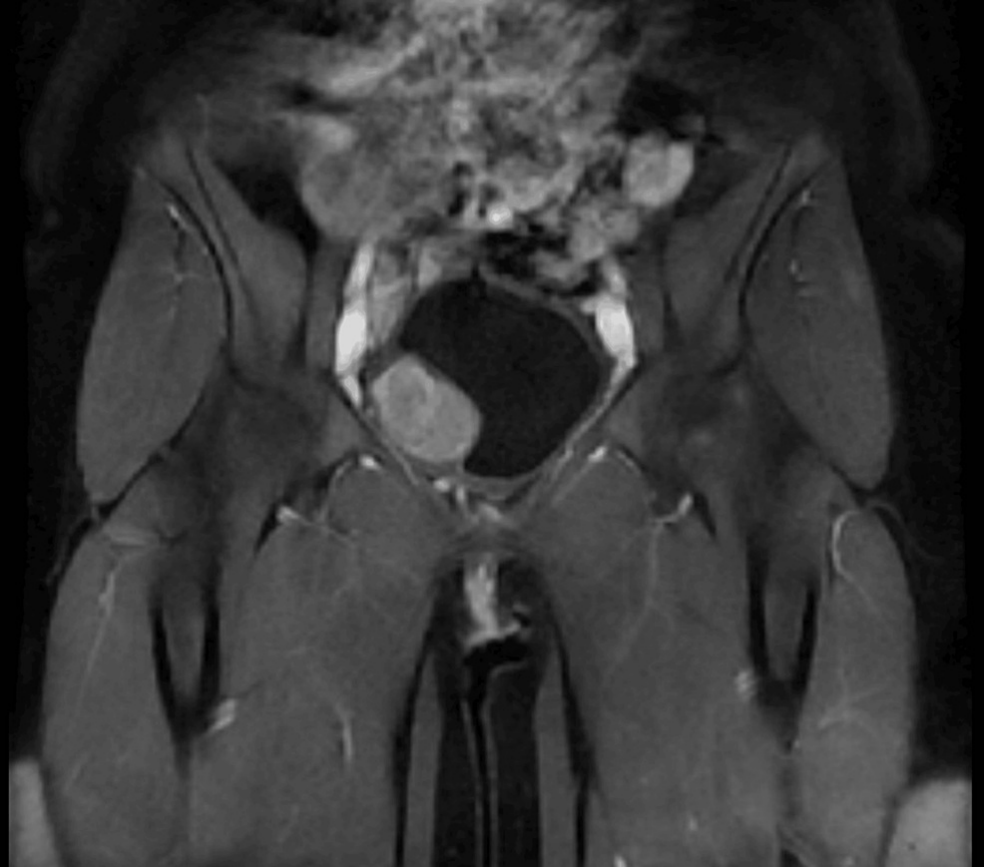
Pre-operative contrast-enhanced pelvic MRI T1 coronal

**Figure 3:**
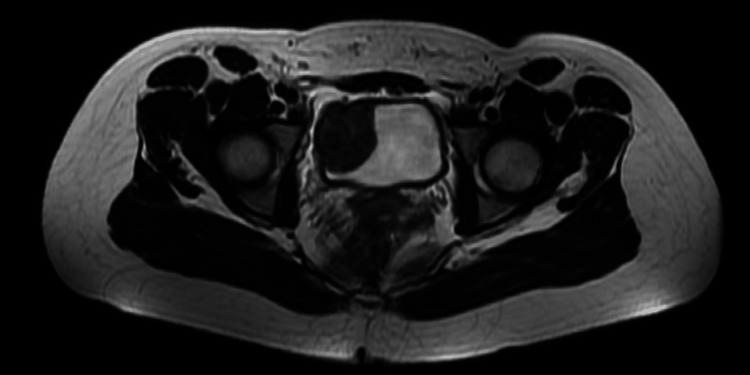
Pre-operative contrast-enhanced pelvic MRI T2 axial

**Figure 4:**
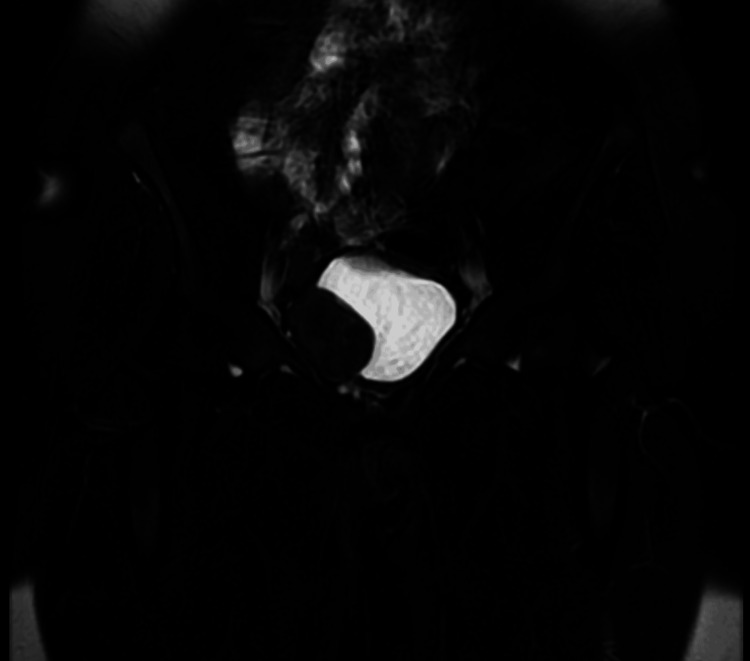
Pre-operative contrast-enhanced pelvic MRI T2 coronal

For further investigation, an abdominopelvic contrast-enhanced MRI was performed which revealed a 30x50 mm fusiform, muscle-originated solid mass. The lesion was isointense on T1 (Figures 1, 2), hypointense on T2-weighted images, and showed an increased contrast enhancement in the post-contrast series (Figures 3, 4). No ureteral orifice invasion or perivesical extension was reported.

**Figure 5:**
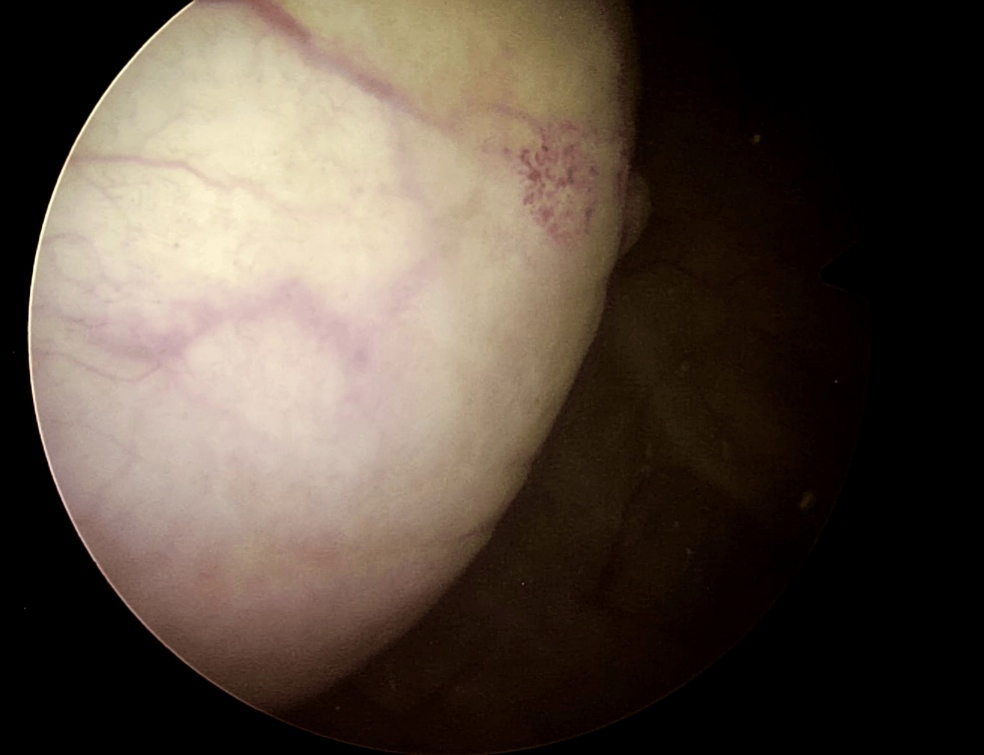
Cystoscopy 5-cm solid mass at the right wall of the bladder which had a normal-looking overlying mucosa.

Cystoscopy under general anesthesia confirmed a 5-cm solid mass at the right wall of the bladder which had a normal-looking overlying mucosa (Figure 5).

**Figure 6:**
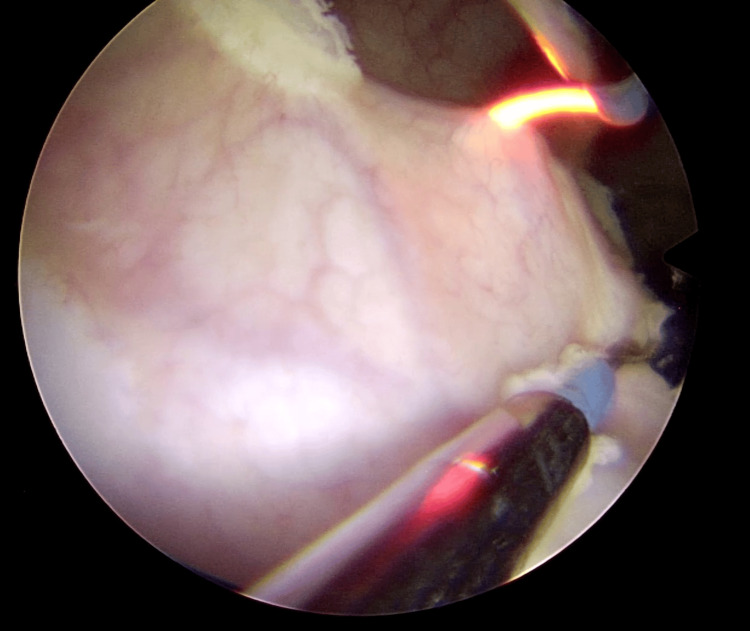
Transurethral piecemeal resection with bipolar energy

**Figure 7:**
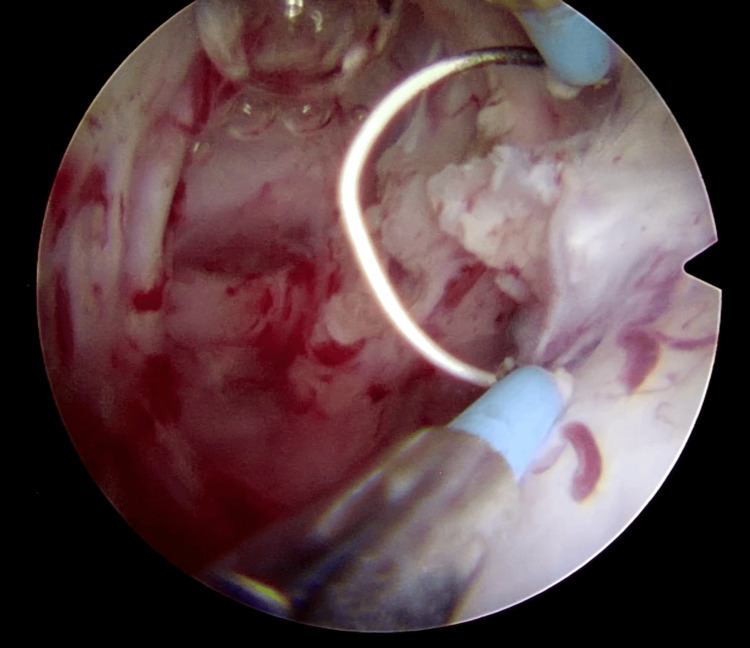
Enucleation of tumor base

Transurethral piecemeal resection with bipolar energy was performed (Figures 6, 7, Video 1).

**Video 1:** Transurethral resection of bladder tumor Cystoscopy under general anesthesia confirmed a 5-cm solid mass at the right wall of the bladder which had a normal-looking overlying mucosa. Transurethral piecemeal resection with bipolar energy was performed. Interestingly no bleeding occurred during the surgery and after complete resection of the tumor. An intact detrusor muscle layer was seen and the base of the tumor and resection margins were fulgurated.

**Figure 8:**
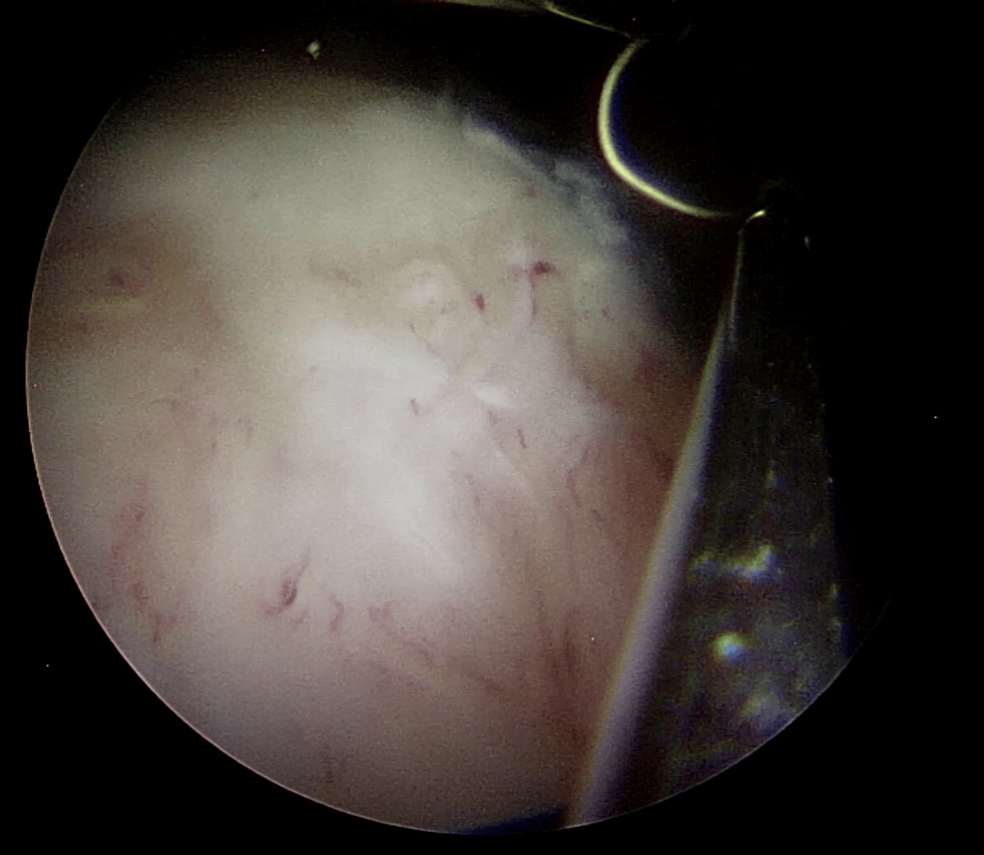
Appearance of enucleated tumor

**Figure 9:**
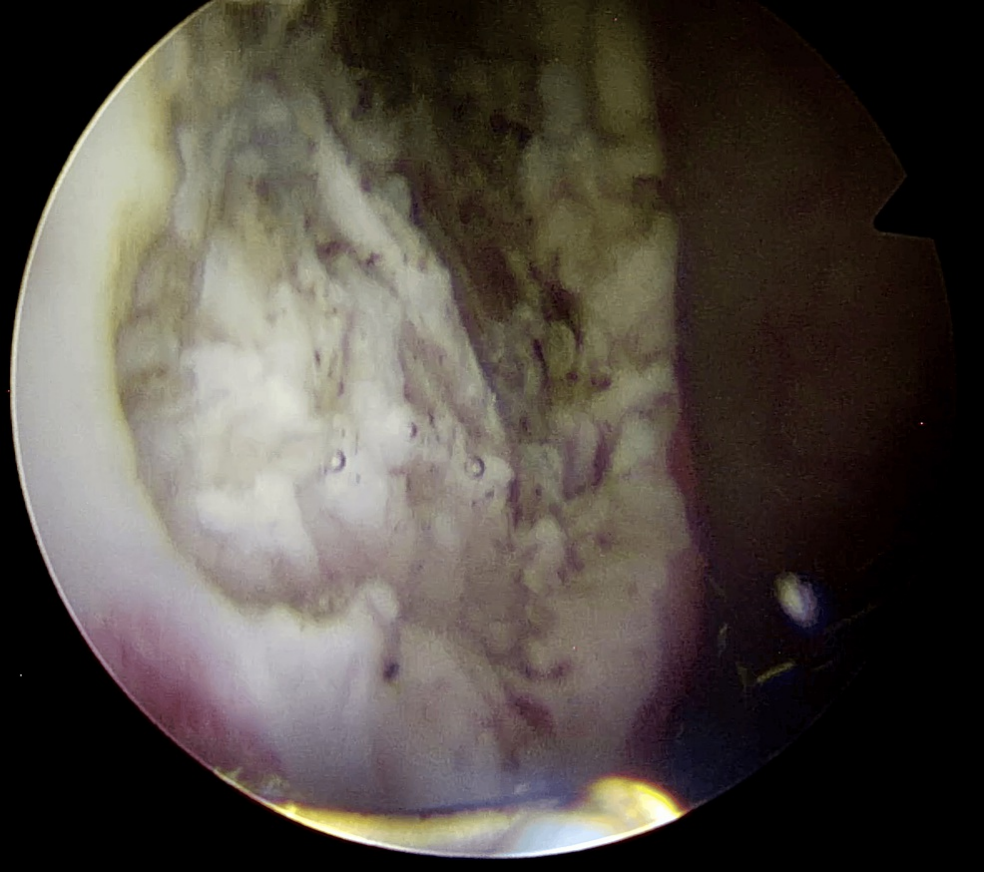
Fulgurated tumor base

Interestingly no bleeding occurred during the surgery and after complete resection of the tumor. An intact detrusor muscle layer was seen and the base of the tumor and resection margins were fulgurated (Figures 8, 9).

A Foley was inserted and continuous irrigation was kept until the postoperative day 1. Foley was removed on the postoperative day 2 and the patient was discharged.

**Figure 10:**
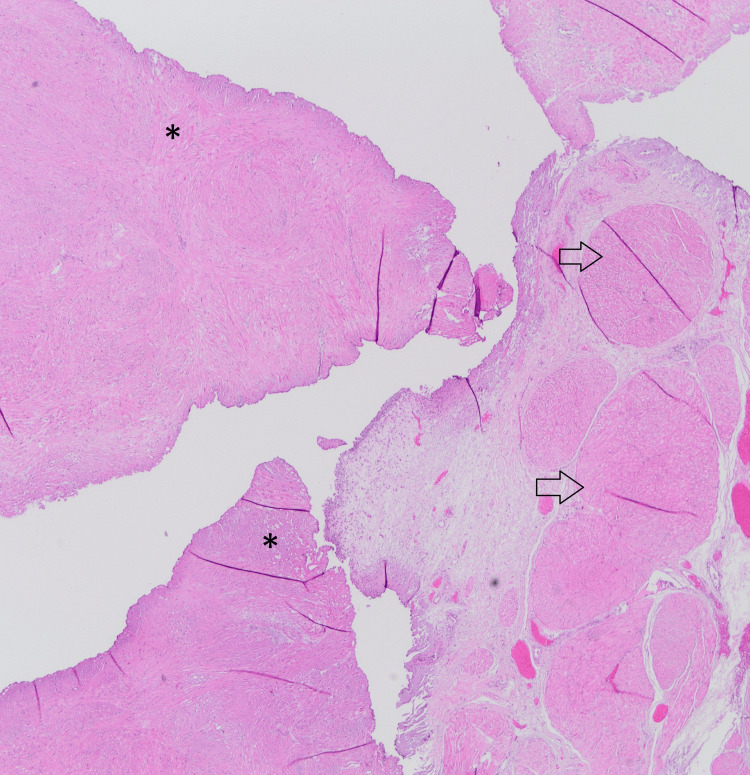
Histopathological examination Transurethral resection revealed numerous tumor fragments composed of spindle cells arranged in a fascicular pattern within the specimen (marked with asterisks), along with a small amount of fragments including normal urothelium and bladder muscle (indicated by arrows).

**Figure 11:**
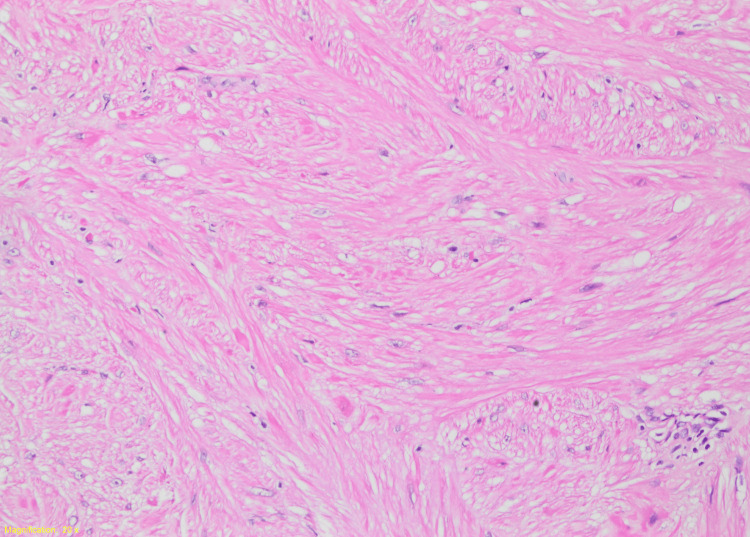
Histopathological examination The tumor consists of spindle uniform cells with low cellularity.

In the macroscopic examination, brown-cream-colored fragmented tissue pieces collectively measuring 5x4x2 cm were observed. Histopathological examination revealed tumor cells in tissue samples with intersecting fascicular architecture, low cellularity, indistinct cytoplasmic borders, eosinophilic cytoplasm, and spindle-shaped nuclei. No pleomorphism, mitosis, or necrosis was observed in the cells (Figures 10, 11).

**Figure 12:**
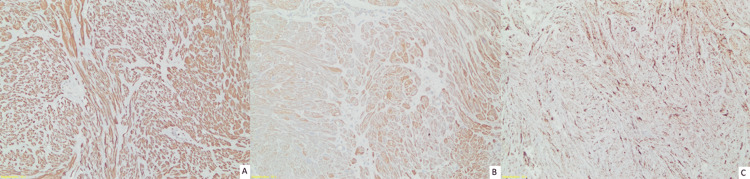
Immunohistochemistry of the specimen with desmin, SMA, FH The tumor cells demonstrate expression for desmin (A) and smooth muscle actin (SMA) (B), as well as fumarate hydratase (FH) (C) (200X).

While the tumor cells express desmin (Figure 12A) and smooth muscle actin (Figure 12B) by immunochemistry, no expression of S100 and CD117 was observed. There was no loss of expression with fumarate hydratase (Figure 12C). Based on these findings, a diagnosis consistent with leiomyoma was rendered for the case.

**Figure 13:**
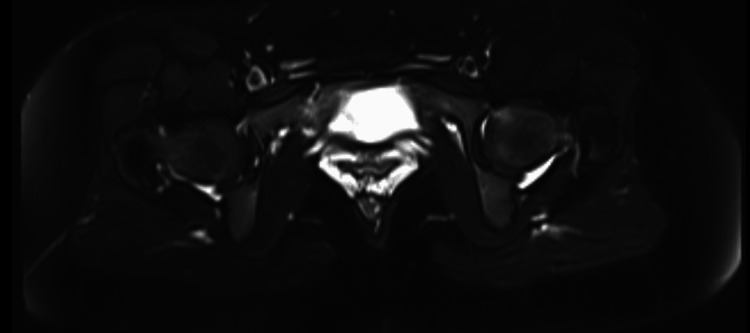
Post-operative contrast-enhanced pelvic MRI T1 axial

**Figure 14:**
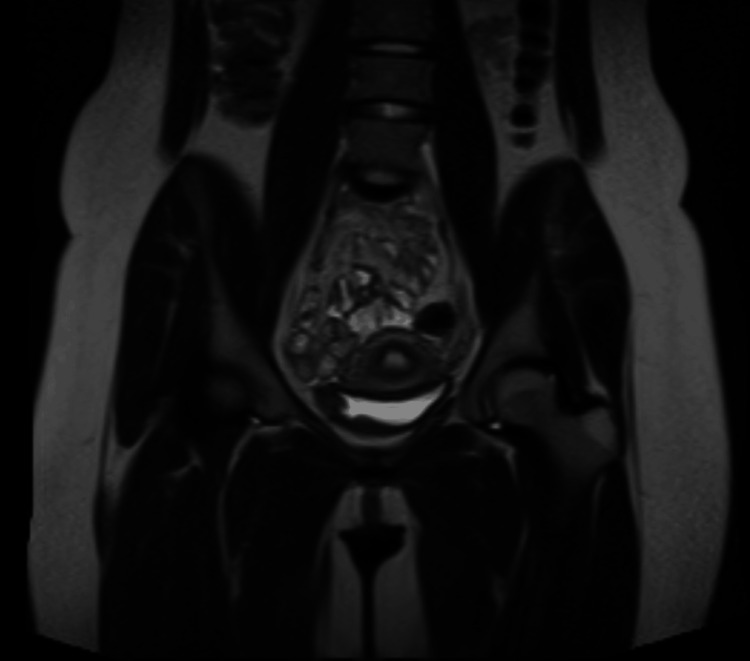
Post-operative contrast-enhanced pelvic MRI T1 coronal

In the postoperative 1st month, an abdominopelvic contrast-enhanced MRI was performed for follow-up, which showed no evidence of recurrence (Figures 13, 14).

## Discussion

In this case, an incidentally diagnosed 15-year-old female patient was successfully treated by a transurethral approach. The bladder mass seemed to be intramural as the overlying mucosa was intact during cystoscopy and complete endoscopic resection was considered to be safe and efficient during the surgery. No complications or recurrence occurred in the postoperative setting.

Compared to other adolescent and pediatric case reports in the literature, in our case, the main symptom was LUTS without haematuria. The explanation for this is believed to be the intramural localisation of the mass, as opposed to the other cases which were intravesical.

The first bladder leiomyoma in children and adolescents was reported by Mutchler and Gorder in 1972 [6]: An 11-year-old female patient presented with haematuria. During cystoscopy, a large polypoid lesion that obstructed the left ureter was observed. Open surgical excision and ureteral reimplantation were performed in the same session and the pathology report confirmed the diagnosis. No complications occurred in the postoperative setting. During the nine-month follow-up, no evidence of tumor was reported.

The second report in the literature was published by Nielsen and Holm in 1976 in Denmark [3]. A 14-year-old female patient who had severe haematuria was presented. As no abstract or full text was available, management and follow-up data are lacking.

In 2005, Calvo et al. reported the third case under the age of 18 years in Spain [14]. In a 17-year-old male patient presenting with haematuria and LUTS, two small bladder lesions were discovered. The patient was treated by transurethral resection. They also reported no recurrence in the postoperative follow-up.

Chen et al. presented a six-year-old male patient in 2012 who had intermittent lower abdominal pain. Upon physical examination, they found a right-sided palpable pelvic mass on the right side. Further ultrasonographic examination showed a hypoechoic mass at the apex of the bladder. A contrast-enhanced CT scan confirmed the 3.2 cm mass. They performed an open partial cystectomy for the case and the pathology report revealed the diagnosis. No complications were reported in the postoperative setting. Follow-up by ultrasound was normal in the postoperative 10th month and the patient was asymptomatic [4].

The last report of pediatric bladder leiomyoma was from India in 2018 published by Mitra et al. [5]. A six-year-old male patient was presented with haematuria and lower abdominal pain. A contrast-enhanced CT scan showed a solid lesion in the left posterolateral wall with extravesicular and posterior urethral extension. Cystoscopic biopsy showed no malignancy (cystitis cystica). Indeed they performed a wide excision of mass that was reported as leiomyoma of the bladder by immunohistochemistry. The postoperative period was event-free and after nine months of follow-up, no evidence of disease was reported.

Differential diagnoses for bladder masses in this age group include urothelial neoplasms, inflammatory myofibroblastic tumors of the bladder, rhabdomyosarcoma, leiomyosarcoma, hemangioma, vascular or neuroendocrine tumors such as paraganglioma or neurofibroma [1].

Radiologic features such as increased contrast uptake, irregular borders, perivesical invasion and heterogenous appearance, and cystoscopic findings such as papillary morphology, and necrotic or inflammatory features may play a role in differential diagnosis, even though it is mandatory to perform a resection of the tumor in order to confirm the final diagnosis with histopathological examination and achieve the cure [1,9,11,15].

Leiomyomas are smooth muscle tumors that are histologically characterized by the presence of fascicles of smooth muscle cells with a moderate to abundant amount of eosinophilic cytoplasm. These tumors do not infiltrate surrounding tissues. The nuclei exhibit an oval to cigar-shaped morphology, with a central location and blunt ends. They do not display any mitotic activity, cellular atypia, or necrosis. Immunohistochemically, the majority of bladder leiomyomas show significant and wide immunoreactivity for smooth muscle actin, muscle-specific actin, desmin, and vimentin, whereas typically absent reactivity of antibodies for cytokeratin and S100 protein [15]. These features are in line with our case and confirm the diagnosis based on current literature.

Both laparoscopic and open surgical techniques for partial or radical cystectomy for bladder leiomyomas were described in the literature previously and no major complications were reported so far. In order to minimize surgical morbidity and increase the quality of life of patients, it is recommended to consider performing transurethral resection of bladder tumors for all small, superficial and resectable tumors. Despite these recommendations, in particular cases with excessive tumor growth or unfavourable localisations and extensions of the tumor, a more radical approach such as open, laparoscopic or robotic partial and even radical cystectomy can be undertaken for treating such tumors [11,16-18].

As previous case reports show no recurrence or metastasis in this age group after treatment, literature for adult patients confirms this statement [1,5,9,10,16,19,20].

## Conclusions

Bladder leiomyomas are uncommon across all age groups, with an even lower incidence in pediatric and adolescent patients. Typical symptoms include obstructive and irritative LUTS and hematuria. Despite its rarity, previous reports suggest that urologists can safely and efficiently manage this condition using transurethral, open, or minimally invasive techniques in appropriately selected patients based on tumor characteristics. To date, no instances of malignant transformation or metastasis have been documented in the literature. In this case, we demonstrate that transurethral resection is a safe and effective treatment for bladder leiomyoma. The major limitation of this case report is the lack of long-term follow-up and cystoscopic surveillance.
